# Promoter DNA Hypermethylation and Gene Repression in Undifferentiated *Arabidopsis* Cells

**DOI:** 10.1371/journal.pone.0003306

**Published:** 2008-10-01

**Authors:** María Berdasco, Rubén Alcázar, María Victoria García-Ortiz, Esteban Ballestar, Agustín F. Fernández, Teresa Roldán-Arjona, Antonio F. Tiburcio, Teresa Altabella, Nicolas Buisine, Hadi Quesneville, Antoine Baudry, Loïc Lepiniec, Miguel Alaminos, Roberto Rodríguez, Alan Lloyd, Vincent Colot, Judith Bender, María Jesús Canal, Manel Esteller, Mario F. Fraga

**Affiliations:** 1 Cancer Epigenetics Laboratory, Molecular Pathology Programme, Spanish National Cancer Centre (CNIO), Madrid, Spain; 2 Plant Epigenetics Laboratory, University of Oviedo, Oviedo, Spain; 3 Cancer Epigenetics and Biology Program (PEBC), Catalan Institute of Oncology (ICO), Barcelona, Catalonia, Spain; 4 Universitat de Barcelona, Facultat de Farmacia, Unitat de Fisiologia Vegetal, Barcelona, Spain; 5 Departamento de Genética, Universidad de Córdoba, Córdoba, Spain; 6 Unité de Recherche en Génomique Végétale, CNRS UMR8114, INRA UMR1165, Université d'Evry Val d'Essonne, Evry, France; 7 Unité de Recherches en Génomique-Info, INRA, Evry, France; 8 Laboratoire de Biologie des Semences, Unité Mixte de Recherche, 204 Institut National de la Recherche Agronomique, Paris-Grignon, Versailles, France; 9 Department of Histology, University of Granada, Granada, Spain; 10 Molecular Cell and Developmental Biology, The Institute for Cellular and Molecular Biology, The University of Texas at Austin, Austin, Texas, United States of America; 11 Department of Biochemistry and Molecular Biology, Johns Hopkins University Bloomberg School of Public Health, Baltimore, Maryland, United States of America; 12 Department of Immunology and Oncology, Centro Nacional de Biotecnología/CSIC, Cantoblanco, Madrid, Spain; Temasek Life Sciences Laboratory, Singapore

## Abstract

Maintaining and acquiring the pluripotent cell state in plants is critical to tissue regeneration and vegetative multiplication. Histone-based epigenetic mechanisms are important for regulating this undifferentiated state. Here we report the use of genetic and pharmacological experimental approaches to show that *Arabidopsis* cell suspensions and calluses specifically repress some genes as a result of promoter DNA hypermethylation. We found that promoters of the *MAPK12*, *GSTU10* and *BXL1* genes become hypermethylated in callus cells and that hypermethylation also affects the *TTG1*, *GSTF5*, *SUVH8*, *fimbrin* and *CCD7* genes in cell suspensions. Promoter hypermethylation in undifferentiated cells was associated with histone hypoacetylation and primarily occurred at CpG sites. Accordingly, we found that the process specifically depends on MET1 and DRM2 methyltransferases, as demonstrated with DNA methyltransferase mutants. Our results suggest that promoter DNA methylation may be another important epigenetic mechanism for the establishment and/or maintenance of the undifferentiated state in plant cells.

## Introduction

The ability of mature plant cells to regenerate a whole organism is probably the most remarkable growth attribute of plant cells that distinguishes them from mammalian cells [1]. The basis of such a capacity in plants lies in the availability of undifferentiated cells that can subsequently differentiate into all the cell types present in a mature organism [2]–[4]. The exact molecular processes involved in the maintenance and/or induction of cell undifferentiation in plants are still poorly understood.

In recent years, the epigenetic mechanisms that control chromatin structure and function, including DNA methylation and histone modification, have emerged as key factors in the regulation of cell growth and differentiation and, thereby, the nuclear reprogramming necessary for dedifferentiation [4]. The presence of a well-defined DNA methylation pattern in plants is necessary for the normal growth and development of the organism [5]–[9]. DNA methylation in plants has two functions: to protect the genome by inactivating transposable elements, and to control the expression of single-copy genes, such as the FWA transcription factor, the BALL pathogen-resistance gene (*BAL*), and the phosphoribosylanthranilate isomerase (*PAI*) family of tryptophan biosynthesis genes (reviewed in [6]). Recent pioneering reports [7], [9]–[11] defining the DNA methylome of *A. thaliana* have reinforced the significance of the two aforementioned tasks of DNA methylation as an epigenetic marker in this model and its relevance as a central coordinator of epigenetic memory.

In mammals, promoter DNA methylation-dependent gene regulation has been found to have important roles in development and differentiation. This is exemplified by the observations that human embryonic stem cells have specific epigenetic signatures [12]–[14], many tissue-specific genes present promoter DNA methylation-dependent regulation (reviewed in [15], [16]), and finally, aberrant hypermethylation-mediated repression of genes involved in cell differentiation results in malignant transformation (reviewed in [17], [18]). In plants, however, there is little information about DNA methylation-dependent mechanisms involved in the control of cell differentiation. Previous work in plants found that the loss of the differentiated state in protoplasts is accompanied by global changes in DNA methylation [19], [20] and histone modification [21] that alter heterochromatin distribution and organization [1], [3], [22], chromatin decondensation [20], [23], and disrupt the nucleolus [19]. Intriguingly, a recent study found that protoplast dedifferentiation is accompanied by a dramatic centromeric heterochromatin decondensation that is not accompanied by changes in DNA methylation or H3K9 dimethylation [24]. With respect to promoter DNA methylation in plants, however, undifferentiation has only been associated with the hypomethylation-dependent upregulation of a few members of the NAC (NAM/ATAF1/CUC2) domain family [19], and there has been no report of genes whose repression by promoter hypermethylation contributes to the maintenance or establishment of the undifferentiated state. To address these gaps in our knowledge by looking for genes regulated by promoter methylation in undifferentiated cells, we adopted two experimental strategies: a genetic approach, using *Arabidopsis* cells deficient in different members of the family of plant DNA methyltransferases, and a pharmacological approach, treating undifferentiated *Arabidopsis* cell suspensions (ACS) with the demethylating drug 5-aza-2′-deoxycytidine (ADC), followed by expression microarray analysis in a similar way to that previously done in whole plants [8]. Using these approaches, we identified three and five hypermethylated genes in callus and cell suspensions, respectively. Promoter hypermethylation primarily occurred at CpG sites and specifically depended on MET1 and DRM2 methyltransferases. Our results suggest that promoter DNA methylation may be another important epigenetic mechanism for the establishment and/or maintenance of the undifferentiated state in plant cells.

## Materials and Methods

### Generation of calluses from DNA methyltransferase KOs and culture conditions

Seeds of *Arabidopsis thaliana* (L.) ecotype Wassilewskija wild type and mutants (*met1*, *cmt3*, *drm2*, *drm2 cmt3*) were surface-sterilized and placed on agar tubes with 10 ml of germination medium containing MS basal medium (mineral salts, vitamins, and micronutrients), 3% sucrose, 0.8% agar, pH 5.8. After stratification at 4°C for 2 days in darkness, seeds were transferred to a growth room and maintained under fluorescent lights (16 h light/8 h dark illumination regime) at 24°C for 10 days. Explants from roots and leaves (approximately 1 cm long) were excised in sterile conditions and sections were put in 60 mm Petri dishes with callus-induction medium (MS basal medium supplemented with 1 mg L^−1^ 2,4- dichlorophenoxyacetic acid (2,4-D), 3% sucrose, 0.8% agar, pH 5.8) at 24°C with a 16∶8 h photoperiod. Nucleic acid was extracted after 20 days of culture once calluses had been established.

Cell suspensions of *Arabidopsis* (Col 0) were obtained as previously described [25]. Cells were grown in Murashige & Skoog (MS) salts medium supplemented with 3% sucrose (w/v), 0.5 mg L^−1^ naphthalene acetic acid, and 0.1 mg L^−1^ kinetin at pH 5.8 in a 16 h light/8 h dark regime at 22°C. For demethylation treatments 5-aza-2-deoxycytidine (5 µM) was added.

### Affymetrix GeneChips

Total RNA was prepared using TRIZOL® (Invitrogen, Carlsbad, CA) and further purified using RNeasy columns (Qiagen, GmbH) according to the manufacturer's instructions. The integrity of RNA was monitored by denaturing agarose gel electrophoresis in 1× MOPS. Biotinylated target RNA was prepared from 5 µg of total RNA using the Affymetrix protocol. Briefly, double-stranded cDNA was prepared from the RNA template using a modified oligo-dT primer containing a 5′ T7 RNA polymerase promoter sequence and the Superscript Choice System for cDNA Synthesis (Invitrogen). cDNA was then used as the template in an *in vitro* transcription reaction. The resulting biotinylated-cRNA “target” was purified on an affinity resin of the GeneChip Sample Cleanup Module Kit (Affymetrix, Santa Clara, CA), randomly fragmented and hybridized on the GeneChip *Arabidopsis* ATH1 Genome Array (Affymetrix). The hybridization reactions were processed and scanned according to the standard Affymetrix protocols. All arrays were globally scaled to a target-intensity value of 600 and then the scaling factor, background, noise, and percentage presence were calculated according to the Affymetrix Data Mining Tool protocols (Affymetrix). All resulting datasets were filtered using the absolute call metric (present or absent) implemented within Microsoft Access (Microsoft Corporation, Redmond, WA). Two biological replicates were done for each sample. The expression profiles for the demethylation treatment and DNA methyltransferase mutants were compared in scatterplots.

### Quantification of global 5-methylcytosine content

5-methylcytosine (5 mC) content was determined by high-performance capillary electrophoresis (HPCE) as previously described [26]. Briefly, genomic DNA samples were boiled, treated with nuclease P1 (Sigma) for 16 h at 37°C, and with alkaline phosphatase (Sigma) for an additional 2 h at 37°C. After hydrolysis, total cytosine and 5 mC content were measured by capillary electrophoresis using a P/ACE MDQ system (Beckman-Coulter). Relative 5 mC content was expressed as a percentage of the total (methylated and non-methylated) cytosine content.

Methylation at CpG sites was quantified as previously described [27]. In brief, DNA was first digested with *XbaI* (20 units µg^−1^) for 2 h at 37°C. For methylation with *SssI*, 5 µl of cut DNA were added to a mixture containing 1 µl Tris-HCl, 1 M (pH 8.0), 2 µl NE-Buffer 2 10× (New England Biolabs), 10 µl S-adenosyl-L-[methyl-^3^H]methionine (14.4 Ci mmol^−1^, 70 µM; Amersham), and 2 µl *SssI* methylase (2 units µl^−1^; New England Biolabs). For methylation with *dam* methylase, the same amount of DNA was added to the following mixture: 1 µl Tris-HCl, 1 M (pH 7.5), 2 µl NE-Buffer for *dam* methylase 10× (New England Biolabs), 0.5 µl water, 10 µl S-adenosyl-L-[methyl-^3^H]methionine, and 1.5 µl *dam* methylase (8 units µl^−1^; New England Biolabs). Both reaction mixtures were incubated for 4 h at 37°C and the incorporation of radioactivity was measured using a 1414 liquid scintillation counter (PerkinElmer, Inc.). The relative percentage of methylated CpG was estimated as [1 – (ratio *SssI*/*d*am/3.2)]×100.

### Analysis of sequence-specific DNA methylation

The methylation status of specific genomic DNA sequences was established by bisulfite genomic sequencing as previously described [28]. Each of these was automatically sequenced in the twelve colonies to measure the methylation status of every single CpG dinucleotide for subsequent statistical analysis. Primers for bilsufite sequencing were designed using Methyl Primer Express Software® (Applied Biosystems). Primer sequences and PCR conditions for methylation analysis are summarized in Table S1.

### Semi-quantitative and quantitative RT-PCR expression analyses

We reverse-transcribed total RNA (2 µg) treated with Dnase I (Ambion) using oligo (dT) 20 primer with ThermoScript TM RT-PCR (Invitrogen). We carried out semi-quantitative PCR reactions in a final volume of 16 µl containing 10× PCR buffer (Ecogen), 50 mM of MgCl_2_, 2 mM of dNTP, 1 µM of each primer and 3 U of EcoStart DNA polymerase (Ecogen). We used 100 ng of cDNA for PCR amplification, and amplified all of the sequences with multiple cycle numbers (25–27 cycles) to determine the appropriate conditions for distinguishing semi-quantitative differences in expression levels. For quantitative RT-PCR analysis, PCR amplifications were performed as followed: 0,20 µg of cDNA, 5 pM of each primer and SYBRGreen PCR Master Mix (Applied Biosystems). Three measurements were analyzed using the Prism 7700 Sequence Detection (Applied Biosystems). Relative quantifications were performed for all genes and *ACTIN* was used as loading control. Primer sequences and PCR conditions for semi-quantitative and quantitative RT-PCR analysis are summarized in Table S1.

### Chromatin immunoprecipitation

Chromatin immunoprecipitation (ChIP) assays were performed as previously described [28] using commercial antiacetylated histones H3 and H4 and antimethylK4 H3 antibodies (Upstate Biotechnologies). Chromatin was sheared to an average length of 0.2–0.5 kb for this analysis. PCR amplification was performed in 25 µl with primers specific to each of the analyzed promoters. The sensitivity of PCR amplification of each promoter was evaluated by serial dilution of total DNA collected after sonication (input fraction). Primer sequences and PCR conditions are summarized in Table S1. All samples were analyzed in triplicate.

## Results

### Genetic identification of putative DNA methylation-dependent transcriptionally repressed genes in *Arabidopsis* callus

**Figure 1:**
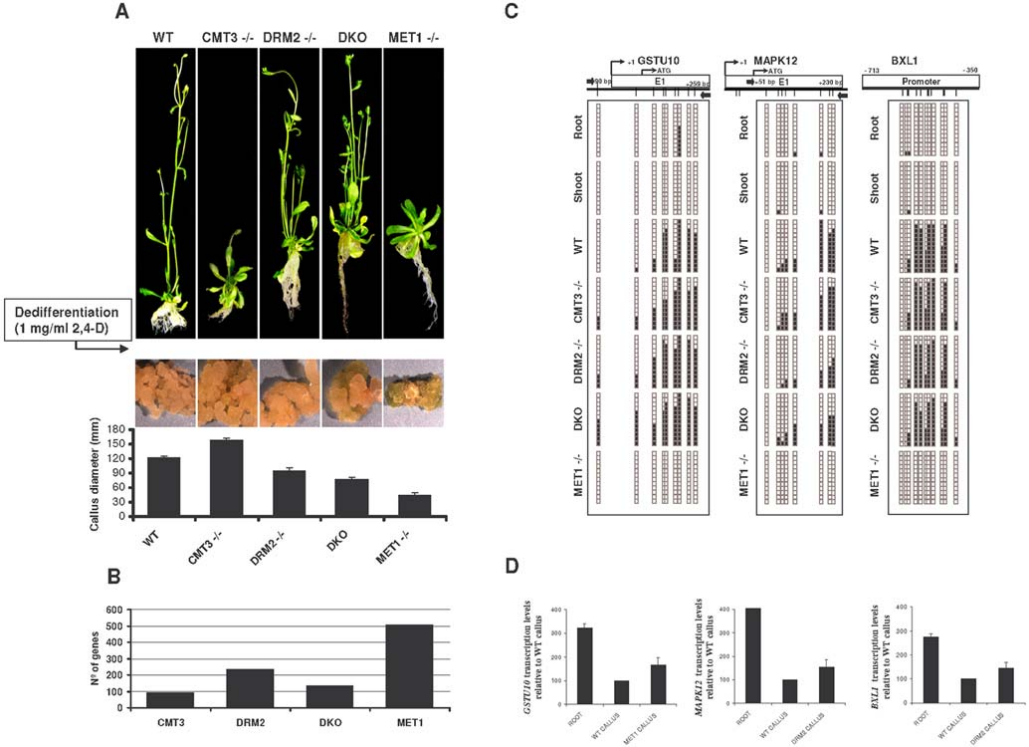
Promoter hypermethylation of *Arabidopsis* callus specifically depends on MET1 and DRM2 methyltransferase activity. (A) Callus induction from DNA methyltransferases mutants. *Upper panels*, morphological aspect of entire plants grown from WT and DNA methyltransferase mutant seeds. *Lower panels*, growth rates of WT and mutant callus generated after treatment with 2,4-D of root explants. (B) Number of candidate genes susceptible to DNA methylation-dependent regulation obtained from each DNA methyltransferase mutant using the two-step criteria described in the Results section. (C) Bisulfite sequencing of twelve individual clones of the *GSTU10*, *MAPK12* and *BXL1* promoters in WT and DNA methyltransferase mutants. (D) Relationship between levels of *GSTU10*, *MAPK12* and *BXL1* expression and promoter DNA hypermethylation. Transcript levels of both genes were analyzed by quantitative RT-PCR and results are expressed as a relative enrichment of the hypomethylated samples (roots and methyltransferase mutants) versus the hypermethylated samples (WT callus).

To look for genes repressed by promoter hypermethylation in callus *Arabidopsis* cells, we used *Arabidopsis* plants deficient in the different plant DNA methyltransferases: *met1* mutant (*MET1* −/−), *cmt3* mutant (*CMT3* −/−), *drm2* mutant (*DRM2* (−/−), and *cmt3/drm2* double mutant (DKO). We produced callus cells from all the mutants using 1 mg L^−1^ 2,4- dichlorophenoxyacetic acid (2,4-D) and measured changes in gene expression relative to differentiated root cells using microarray chips containing around 22,500 transcripts (*Arabidopsis* ATH1 Genome array 1 GENECHIP® AFFYMETRIX®) (Figure 1A). A sequence of criteria was used to select genes dependent on each DNA methyltransferase. First, genes downregulated in the WT callus relative to WT plants were identified in order to select putative promoter hypermethylation-dependent undifferentiation-associated genes. We used the U rank-statistic test, which ignores the magnitude of the expression values but distinguishes samples with higher and lower levels of expression. The statistic was determined for each comparison and genes with values of U = 0 were selected. A fold-change value, given by the ratio of the means of the two comparison groups was calculated, and genes with at least twice the average expression in WT plants as in WT callus were selected. Second, from the selected genes, we picked those genes that were upregulated (according to the two-step criteria described above) in callus of each DNA methyltransferase mutant relative to the WT callus in order to associate gene regulation with a specific plant DNA methyltransferase. Third, of the candidate genes obtained under the previous criteria, we discarded those common to at least two mutants. Using these criteria we identified 96 genes for *cmt3*, 235 for *drm2*, 134 for the double mutant DKO, and, strikingly, 505 for the *met1* mutant (Figure 1B). This may be evidence of a role for MET1 methyltransferase in promoter hypermethylation in *Arabidopsis* callus cells. The observation is also consistent with the fact that callus induction was less efficient using cells from the *met1* mutant. Callus from MET1 deficient cells presented low proliferation rates and maintained differentiation-associated features like green pigmentation (Figure 1A).

To determine whether promoter hypermethylation is involved in the selective repression of all DNA methyltransferase-specific loci, we investigated the genomic DNA methylation status of the promoter region of twenty candidates for the *met1* mutant, one candidate for the *cmt3* mutant, and nine candidates for the *drm2* mutant (Table S2). The number of genes selected for each mutant was proportional to the number of candidates identified with each mutant. The selection of the 30 genes from the total of 836 candidates identified with the three DNA methyltransferase mutants was made on the basis of their putative role in cell differentiation and cell division (Table S3). We designed specific primers for DNA modified with sodium bisulfite around the transcription start site of each gene (Table S1). Of the 30 genes analyzed, three, *GSTU10* (At1g74590), *MAPK12* (At2g46070) and *BXL1* (At5g49360), presented a higher methylated cytosine content in the CpG sites of the WT callus. We studied the origin of the CpG hypermethylation and compared the degree of DNA methylation in calluses from WT and methyltransferase mutants. We observed a strong decrease of CpG methylation levels in *met1* mutants but not in *cmt3* and *dko* callus (Figure 1C). *drm2* mutants also presented a decrease of CpG methylation within the promoter region of *MAPK12* (Figure 1C). As expected, differentiated tissues such as roots, shoots or leaves from WT plants were completely unmethylated (Figure 1C). The remaining 27 selected genes were completely unmethylated in all the tissues analyzed (Figure S1). Interestingly, promoter hypermethylation of *GSTU10*, *MAPK12* and *BXL1* was associated with gene repression (Figure 1D). These results suggest that hypermethylation of *GSTU10*, *MAPK12* and *BXL1* in WT callus results from a MET1/DRM2-dependent mechanism. *MAPK12*, which was initially identified as a DRM2-dependent gene, was less methylated in *drm2* mutants than in WT callus, but the *drm2* mutant retained several methylated CpGs. Moreover, the *met1* mutant completely failed to hypermethylate the *MAPK12* promoter after callus induction, which suggests that even though DRM2 might be important for establishing dedifferentiation-associated promoter hypermethylation, MET1 could be more important still.

### Pharmacological identification of putative DNA methylation-dependent transcriptionally repressed genes in *Arabidopsis* cell suspensions

**Figure 2:**
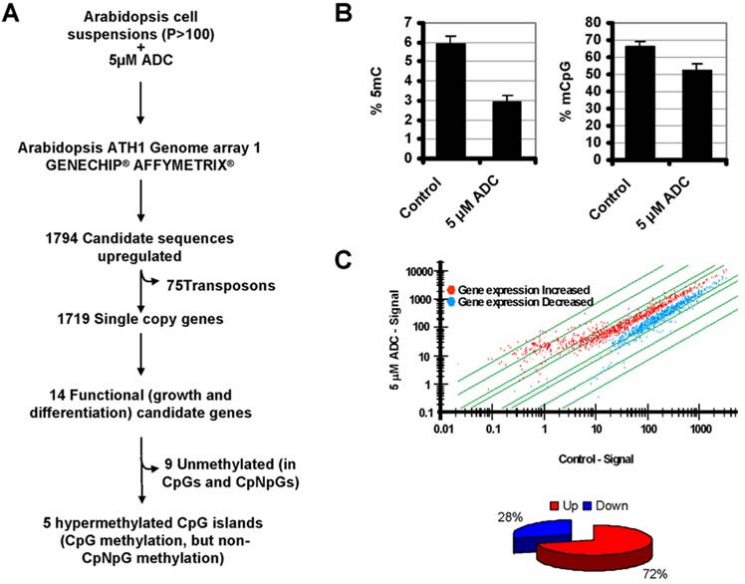
Reactivation of hypermethylated genes in *Arabidopsis* cell suspensions (ACS) using demethylating drugs. (A) Flowchart for identification of hypermethylated growth-associated genes. We used *Arabidopsis* cell suspensions after 5 µM ADC treatments followed by cRNA hybridization to a 22,500-oligonucleotide microarray. We obtained over 1,794 unique sequences overexpressed after treatments. Of these, 1,719 corresponded to known genes and 75 to repeat elements. We selected fourteen genes to test for promoter hypermethylation by direct bisulfite sequencing; five of them were found to be methylated in *Arabidopsis* cell suspensions but not in differentiated tissues such as roots and shoots. (B) Quantification of DNA methylation as: overall 5-methyl-cytosine (5 mC) using high-performance capillary electrophoresis (*Left panel*), and percentage of methylated CpGs using the methyl acceptor assay (*Right panel*). (C) Reactivation of genes in *Arabidopsis* cell suspensions after treatment with the demethylating drug ADC. *Upper panel*, scatterplots showing expression profiles of control cells and cells treated with ADC obtained by Affymetrix GeneChip technology; *Lower panel*, relative percentage of overexpressed and repressed genes after treatment with ADC.

As a second approach to identify genes repressed by promoter DNA methylation in undifferentiated *Arabidopsis* cells we generated *Arabidopsis* cell suspensions by *in vitro* culture of seedling-excised roots in the presence of auxins and cytokinins [25]. We then used the demethylating agent ADC (5 µM) for 4 days to reactivate putative genes epigenetically silenced in the *Arabidopsis* cell suspensions (Figure 2A). The efficiency of the DNA demethylating treatment was measured by HPCE [26]. This revealed a 51.3% relative decrease in global DNA methylation (Figure 2B, left). We also used the methyl-acceptor assay [27] and found that 21.2% of the cytosines within the dinucleotide CpG became unmethylated after treatment with the DNA demethylating drug (Figure 2B, right).

After ADC treatment, we measured changes in gene expression using the same microarray chips as described in the previous section. To detect genes with significantly higher expression in ADC-treated cells than in wild-type cells, and to take both magnitude and rank into account, we used the two-step criteria described above. Analysis of the expression microarray data revealed that changes in expression after treatment with the demethylating drug involved the overexpression of genes in most cases (71.9%, 1,794 genes with U = 0 and at least twice the average level of expression) (Figure 2C). Of the 1,794 candidate sequences upregulated after treatment with the DNA demethylating agent, 1,719 corresponded to single-copy genes and 75 to transposons (Figure 2A). A substantial number of transposable elements (11/75) belonged to the CACTA-like transposase family [29].

### Promoter hypermethylation and transcriptional silencing of single-copy genes in *Arabidopsis* cell suspensions

**Figure 3:**
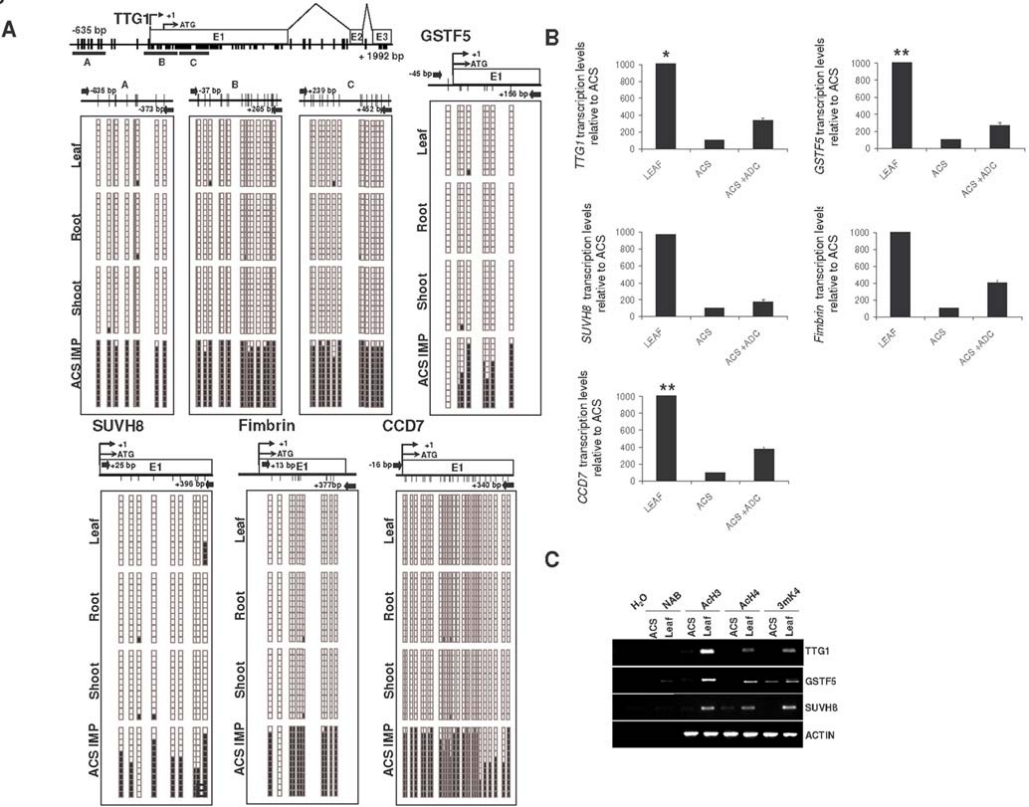
Genomic DNA methylation status of reactivated genes in leaf and *Arabidopsis* cell suspensions (ACS). (A) Bisulfite genomic sequencing of twelve clones of the *TTG1* (three different regions), *GSTF5*, *SUVH8*, *Fimbrin* and *CCD7* promoters. In the schematic representations of the methylation status of each CpG dinucleotides black and white dots indicate methylated and unmethylated CpGs, respectively. ACS IMP: *Arabidopsis* cell suspensions at intermediate passage. (B) Expression profiles of reactivated genes in leaves and *Arabidopsis* cell suspensions determined by quantitative RT-PCR assays. *TTG1*, *GSTF5*, *SUVH8*, *Fimbrin* and *CCD7* are more strongly expressed in leaf tissues. Expression in cell suspensions can be restored by treatments with the demethylating drug ADC. ACTIN was used as a control. (*) the expression level of *TTG1* in leafs is 80-fold higher than in ACS. (**) the expression level of *GSTF5* and *CCD7* in leafs is 40-fold higher than in ACS. (C) Promoter CpG island hypermethylation is associated with changes of histone modifications. Chromatin immunoprecipitation analysis of the histone-modification status of the promoters of the *TTG1*, *GSTF5*, and *SUVH8* genes. NAB is the control without antibody. The promoter region of ACTIN is used as a control. AcH3, acetylated histone H3; AcH4, acetylated histone H4; 3mK4, trimethyl-lysine 3 histone H3.

**Figure 4:**
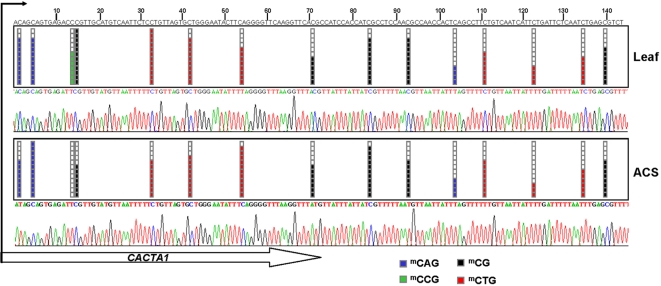
Bisulfite genomic sequencing of twelve individual clones of the repeat DNA CACTA in leaves and *Arabidopsis* cell suspension (ACS). Black and white dots indicate methylated and unmethylated CpGs, respectively. Methylation at CAG, CG, CCG, and CTG sites is represented by blue, black, green, and red boxes, respectively. The lower panels illustrate representative electropherograms.

We analyzed the possible DNA methylation-associated silencing of the fourteen selected single-copy genes by bisulfite genomic sequencing of multiple clones using primers located around the transcriptional start site of each candidate gene (Table S4). We selected these genes using a dual criterion: genes statistically upregulated after the treatment with ADC but, at the same time, with a role in growth and differentiation (Table S3). In the *Arabidopsis* cell suspensions we observed dense DNA hypermethylation around the transcription start site of five genes: *TTG1* (At5g24520), *GSTF5* (At1g02940), *SUVH8* (At2g24740), fimbrin (At2g04750) and CCD7 (At2g44990) (Figure 3A). The nine remaining sequences analyzed were completely unmethylated (Figure S2). DNA methylation of *TTG1*, *GSTF5*, *SUVH8*, *fimbrin* and *CCD7* in the cell suspensions was found mostly in the CpG dinucleotide, and not in the CpNpG and CpNpN motifs. The same regions of these five genes were completely unmethylated in differentiated tissues of *Arabidopsis*, such as leaf, root, and shoot (Figure 3A). It is worth considering the difference in the DNA methylation status of the aforementioned five single-copy genes and the transposons in our *Arabidopsis* system. While we found that *TTG1*, *GSTF5*, *SUVH8*, *fimbrin* and *CCD7* presented specific CpG hypermethylation in the cell suspensions, but were fully unmethylated in all the differentiated tissues studied, the CACTA-like transposons (At1g43840, At2g13160, At2g12980) exhibited dense methylation of both CpG and CpNpG motifs independently of their tissue differentiation status (Table S5 and Figure 4). These results highlight the distinctive and specific CpG hypermethylation events occurring in the promoter region of single-copy genes and imply that dedifferentiation-associated promoter hypermethylation in *Arabidopsis* depends on MET1 and DRM2 methyltransferases.

The promoter hypermethylation of *TTG1*, *GSTF5*, *SUVH8*, *fimbrin* and *CCD7* was always associated with transcriptional silencing, and expression could be restored with the use of the DNA demethylating agent ADC (Figure 3B). Chromatin immunoprecipitation analysis with antibodies raised against histone modifications associated with transcriptional activation [30] demonstrated a loss of acetylated histones H3 and H4 and trimethylated lysine 4 histone H3 within all of the hypermethylated promoters (*TTG1*, *GSTF5*, and *SUVH8*) in cell suspensions compared with differentiated tissues (Figure 3C).

In this model of *Arabidopsis* cell suspensions, the undifferentiated and highly proliferative state is known to depend on the presence of phytohormones (NAA and Kinetin) in the culture medium [31]. For this reason, we wondered whether their removal might lead, in addition to the differentiation and cell growth arrest described herein (Figure S3A), to the loss of promoter hypermethylation. Bisulfite genomic sequencing showed that depletion of just one of the phytohormones was sufficient to induce a loss of CpG methylation in all three promoters studied (Figure S3B). To asses whether changes in phytohormone- induced DNA methylation were associated with changes in *MET1* expression we analyzed the levels of MET1 transcripts by quantitative RT-PCR in *Arabidopsis* cell suspensions exposed or not to NAA and/or Kinetin. Our results showed that removal of any of the phytohormones, did not result in any significant change in *MET1* expression (Figure S3C) which suggest that other mechanisms such as control of its enzymatic activity or recruitment to specific promoters could be involved.

To establish whether the specific genes that become hypermethylated in dedifferentiated cells depend on the type of phytohormone used to maintain the undifferentiated state, we compared the DNA methylation status of two hypermethylated genes in callus cells obtained with 2,4-D (*GSTU10* and *MAPK12*) and the *Arabidopsis* cell suspensions maintained with kinetin and NAA. We found that *GSTU10* and *MAPK12* were also densely methylated in the *Arabidopsis* cell suspensions (Figure S4). In contrast, the promoter region of three genes identified in the well-established *Arabidopsis* cell suspensions (*TTG1*, *GSTF5*, and *SUVH8*) remained unmethylated in the early 2,4-D-induced callus (Figure S5). The lack of promoter hypermethylation of *TTG1*, *GSTF5*, and *SUVH8* genes in cells soon after the induction of callus prompted us to question whether the hypermethylation-mediated repression of these three genes is indispensable to the maintenance of the undifferentiated state. To address this, we monitored the promoter hypermethylation of the *TTG1* gene in *Arabidopsis* cell suspensions of different passage number. Intriguingly, we found a drop in CpG island methylation levels of the *TTG1* gene after 400 passages (Figure S6), in a similar manner to that described for other methylated DNA sequences in mammals [32]. This finding, in conjunction with the unmethylated status of *TTG1* in callus, suggest that other mechanisms apart from DNA methylation might be involved in the regulation of *TTG1* in undifferentiated Arabidopsis cells.

## Discussion

Promoter DNA methylation-dependent gene regulation in mammalian cells has important roles during development and differentiation. For example, human embryonic stem cells have specific epigenetic signatures [12]–[14], many tissue-specific genes present promoter DNA methylation-dependent regulation [15], [16], and, finally, aberrant hypermethylation-mediated repression of genes involved in cell differentiation results in malignant transformation [17], [18]. In addition to hypermethylated genes, undifferentiated mammalian cells also feature genes that are hypomethylated with respect to their differentiated counterparts [33]. In plants, cells acquiring pluripotency have been described as exhibiting hypomethylation-dependent upregulation of several members of the NAC (NAM/ATAF1/CUC2) domain family [19]. In the present study we have demonstrated that *Arabidopsis* calluses and cell suspensions can use promoter DNA methylation to repress specific single-copy genes that are unmethylated and expressed in differentiated cells from various origins. However, it is important to state that this epigenetic mechanism is not a general response to cell culture as just a minor fraction of the genes become hypermethylated in callus and cell suspensions.

We found that promoters of the *MAPK12*, *GSTU10* and *BXL1* genes are densely hypermethylated in callus and cell suspensions, whilst the *TTG1*, *GSTF5* and *SUVH8* genes become occasionally hypermethylated only in cell suspensions. Interestingly, the role of TTG1 in cell fate and differentiation is very well documented [34]–[38]. Thus, as TTG1 plays an important role in plant development, its methylation-dependent repression in undifferentiated *Arabidopsis* could directly contributes to the manteinance of the undifferentiated status. However, it is important to bear in mind that although recovery of TTG1 activity in cell suspension results in a modest induction of cell differentiation (data not shown), our discovery of *in vitro* cultured cells lacking *TTG1* promoter hypermethylation indicates that the regulation of *TTG1* in the establishment and/or maintenance of the undifferentiated state is a complex process that can sometimes include promoter DNA methylation.

Intriguingly, we found that promoter hypermethylation in cell suspensions and callus primarily occurs at CpG but not at CpNpG or CpNpN sites. The absence of CpNpG and CpNpN methylation at the promoter region of genes hypermethylated specifically in cell suspensions and callus suggests that the process depends primarily on the plant MET1 methyltransferase. This is consistent with the facts that *Arabidopsis met1* mutants are more resistant to producing callus than are control plants, and that the number of hypermethylated genes after callus induction that we identified by genetic identification was lower in MET1- and DRM2-deficient plants than in controls and *cmt3* mutants. Moreover, these results suggest that, at least in *Arabidopsis* cell suspensions and callus, promoter-specific CpG methylation may be important for the regulation of the expression of some genes.

**Figure 5:**
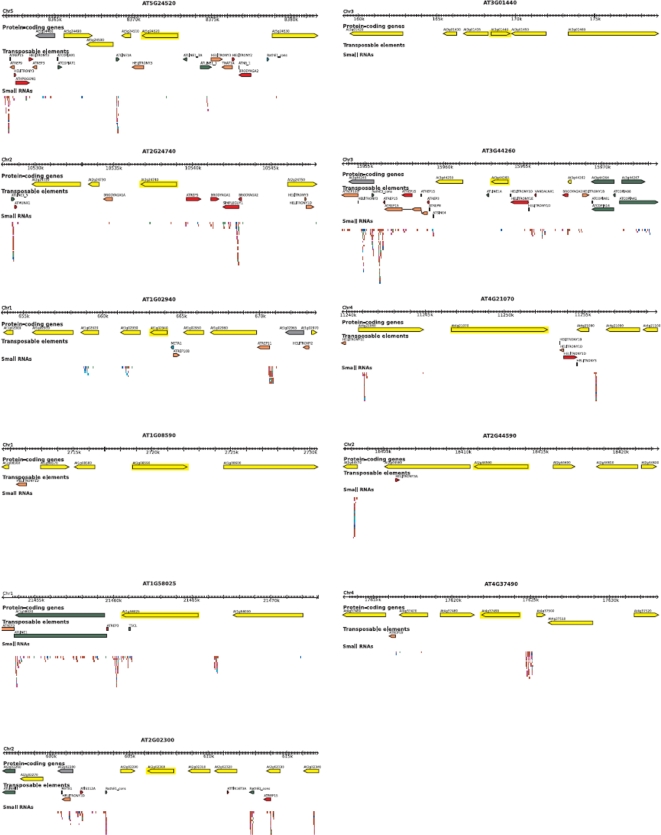
Gbrowse view of gene, transposable element and small RNA annotation of 20 kb segments centered on the 2 CpG island containing genes selected with the genetic (*GSTU10* and *MAPK12*) and pharmacologic (*TTG1* and *SUVH8*) approaches. Gene annotation is from TAIR (v7). TE annotation was performed using a novel detection pipeline (HQ, NB, and VC, manuscript in preparation). Small RNA data were directly imported from the ASRP database (http://asrp.cgrb.oregonstate.edu/cgi-bin/gbrowse/thaliana-v5) and corresponded to deep sequencing of small RNAs extracted from wild type plants (seedlings, leaves, and flowers).

The way of how undifferentiation-associated hypermethylation is established and maintained remains unclear. As MET1 is thought to be important in the silencing of repeated transposable elements [7], one possibility is that MET1 is specifically recruited to repeated DNA associated with its target promoters. Indeed, the five genes for which we found CpG hypermethylation by pharmacological identification contained TEs within 1 kb of the start codon, whereas only one gene (At3g44260) of the remaining eight selected had TE insertions at its 5′ end, suggesting the involvement of TEs in cell suspensions-associated methylation (Figure 5). Intriguingly, none of the genes identified by the genetic approach contained TE insertions around the transcription start point (Figure 5), which suggests that there may be different mechanisms by which DNA methylation is established in the various undifferentiated cells. However, as our study is centered in a limited number of genes, further genome-wide studies are need to asses this issue. Another possibility is that DRM2 is directed to specific promoters through iRNA-mediated mechanisms for genes hypermethylated early upon callus induction [7], [39]. However, *drm2* mutants retain CpG methylation, which suggests that MET1 must also be involved. It can also be possible that MET1 expression depends on DRM2 but it is unlikely as drm2 mutants and WT callus present similar levels of MET1 expression (Figure S7).

Apart from the single-copy genes, we observed that the treatment of *Arabidopsis* cell suspensions with the demethylating drug ADC also induced the overexpression of 75 transposons. However, considering that plant transposable elements are known to be densely methylated [6], the number of transposable elements reactivated (75/1,794) was much lower than expected. This might have been because transposable elements can become hypomethylated and reactivated under stress conditions [40], which would preclude their reactivation with demethylating drugs. Consistent with such an explanation, although the transposable elements identified in this study had lower DNA methylation levels in the cell suspensions than in leaves, they retained sufficient methylation in response to the treatment with the demethylating drug. Given that most of the genomic DNA methylation is contained in repeated heterochromatic DNA [7], *in vitro* induction of the undifferentiated state could imply an overall decrease of genomic DNA methylation, except for the case of promoter hypermethylation. A scenario where a gene becomes hypermethylated in the context of global DNA hypomethylation has previously been reported for SUPERMAN [41], AGAMOUS [42], and Bonsai [43] genes.

Dedifferentiation in plants has previously been associated with promoter hypomethylation of certain genes [19]. Thus, in conclusion, we propose that callus and *Arabidopsis* cell suspensions possess a unique epigenetic signature with subsets of genes whose expression is controlled by promoter hypomethylation and hypermethylation, as has been described in mammals [33]. Further research is needed to determine the exact functional role of gene regulation by promoter DNA methylation during plant cell differentiation, and in the establishment and maintenance of the undifferentiated state.

## Supporting Information

Figure S1 Bisulfite genomic sequencing of twelve individual clones of nine representative genes unmethylated in leaves and Arabidopsis wild type callus. Black and white dots indicate methylated and unmethylated CpGs, respectively. Lower panels, representative electropherograms.
(0.05 MB PDF)

Figure S2 Bisulfite genomic sequencing of twelve individual clones of six representative genes unmethylated in leaves and Arabidopsis cell suspensions (ACS). Black and white dots indicate methylated and unmethylated CpGs, respectively. Lower panels, representative electropherograms.
(0.04 MB PDF)

Figure S3 Promoter hypermethylation in cell suspensions is dependent on combined phytohormone action and is associated with histone hypoacetylation and gene repression. Bisulfite genomic sequencing of the TTG1, GSTF5, and SUVH8 promoters in the presence or absence of the demethylating drug ADC, and the phytohormones NAA and kinetin in Arabidopsis cell suspensions. (A) Quantification of growth rates and the relative percentage of methylated CpGs. (B) Schematic representations of the methylation status of each CpG dinucleotide. Black and white dots indicate methylated and unmethylated CpGs, respectively. (C) Analysis of MET1 expression in Arabidopsis cell suspensions (ACS) growing in normal culture medium, in presence or in absence of phytohormones (kinetin or NAA). Transcript levels were analyzed by quantitative RT-PCR and results are expressed as a value relative to the expression in control ACS.
(0.04 MB PDF)

Figure S4 Promoter DNA methylation of MAPK12 and GSTU10 genes was also analyzed after different numbers of passages in Arabidopsis cell suspensions (ACS): IMP, intermediate passages; LP, late passages. Black and white dots indicate methylated and unmethylated CpGs, respectively.
(0.02 MB PDF)

Figure S5 Bisulfite genomic sequencing of twelve individual clones of TTG1 (A), GSTF5 (B), and SUVH8 (C) genes in cell suspensions derived from WT and DNA methyltransferase mutants of Arabidopsis thaliana (L.). Black and white dots indicate methylated and unmethylated CpGs, respectively.
(0.03 MB PDF)

Figure S6 Bisulfite genomic sequencing of twelve clones of the TTG1 (three regions) 5′-regulatory region in Arabidopsis cell suspensions at 300, 330, and 400 passages. Schematic representations of the methylation status of each CpG dinucleotide. Black and white dots indicate methylated and unmethylated CpGs, respectively.
(0.03 MB PDF)

Figure S7 Transcription levels of MET1 in WT callus and drm2 mutant. Transcript levels were analyzed by quantitative RT-PCR and results are expressed as a value relative to the expression in WT callus.
(0.01 MB PDF)

Table S1 Primer sequences and annealing temperatures.
(0.02 MB PDF)

Table S2 Genetic identification of methylated genes in dedifferentiated Arabidopsis cells.
(0.02 MB PDF)

Table S3 List of candidate genes selected for validation with bisulfite sequencing analysis.
(0.03 MB PDF)

Table S4 List of Arabidopsis thaliana genes upregulated after treatment with ADC.
(0.02 MB PDF)

Table S5 Sequence context of some of the DNA methylation found in Arabidopsis cell suspensions and leaves.
(0.01 MB PDF)
