# Supplementary figures and images for: Promoter DNA Hypermethylation and Gene Repression in Undifferentiated Arabidopsis Cells
Source: PLoS One. 2008 Oct 1;3(10):e3306. doi: 10.1371/journal.pone.0003306 (PMC2556100; doi:10.1371/journal.pone.0003306)

Figure S1

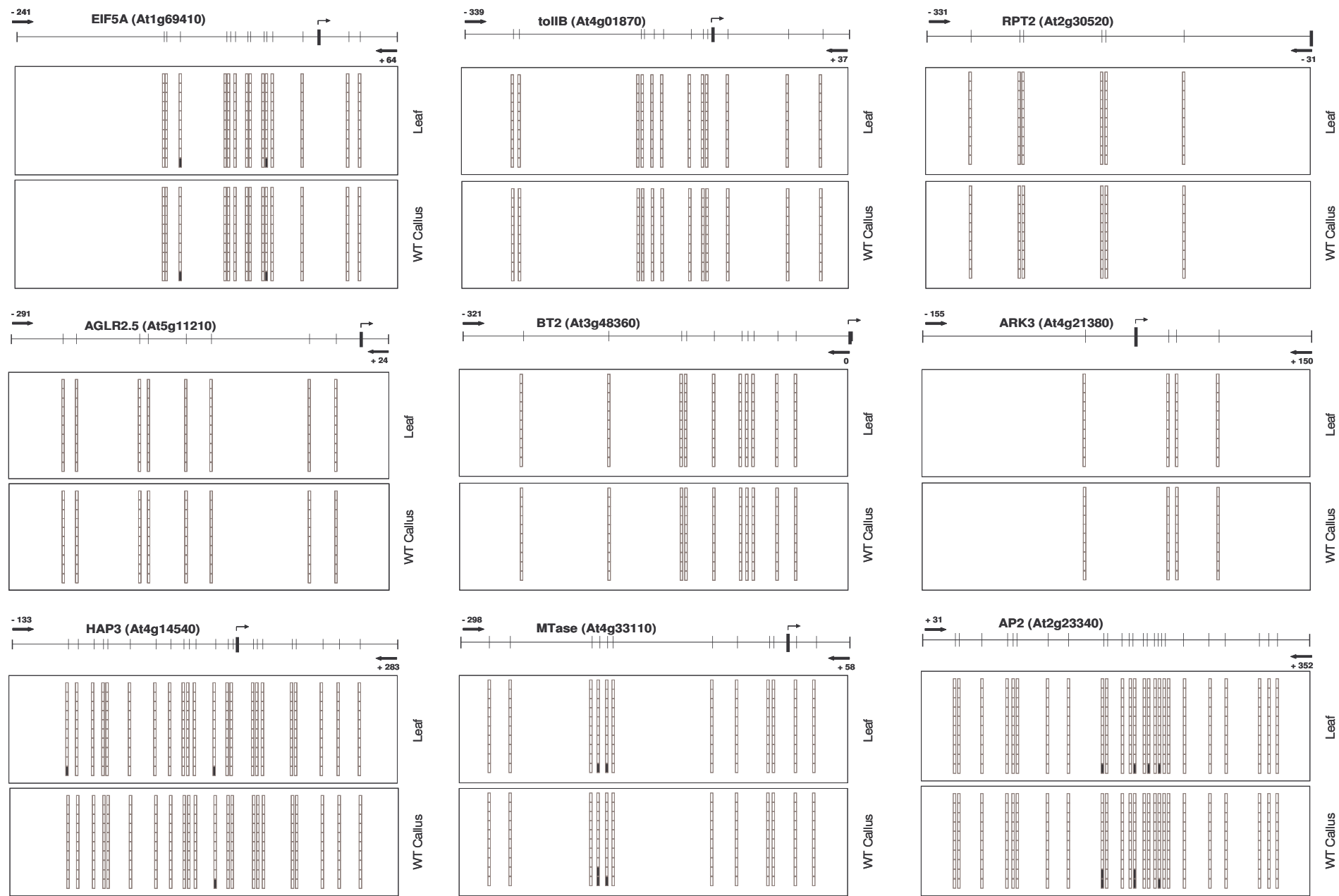

Supplement: Figure S1 — Bisulfite genomic sequencing of twelve individual clones of nine representative genes unmethylated in leaves and Arabidopsis wild type callus. Black and white dots indicate methylated and unmethylated CpGs, respectively. Lower panels, representative electropherograms. (0.05 MB PDF) [file pone.0003306.s001.pdf]

Figure S2

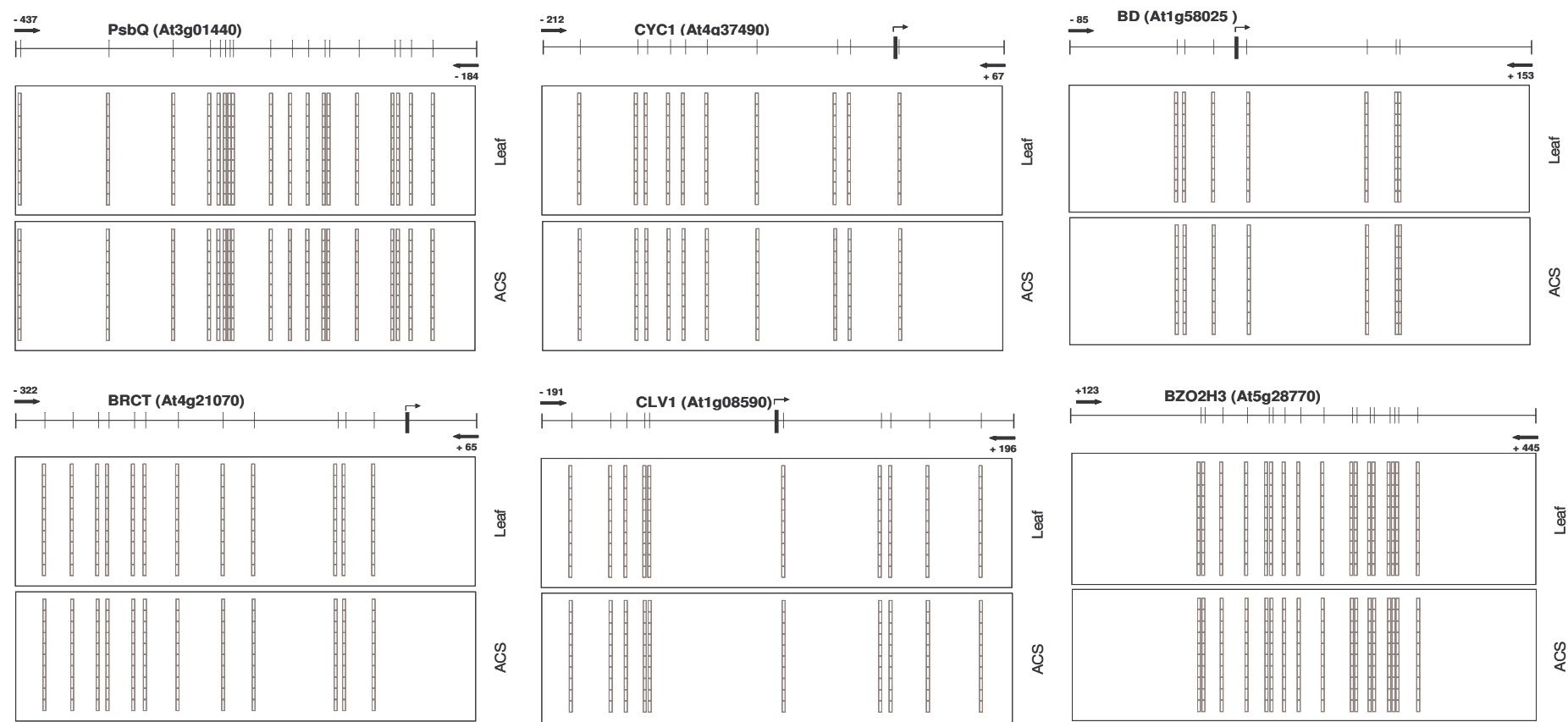

Supplement: Figure S2 — Bisulfite genomic sequencing of twelve individual clones of six representative genes unmethylated in leaves and Arabidopsis cell suspensions (ACS). Black and white dots indicate methylated and unmethylated CpGs, respectively. Lower panels, representative electropherograms. (0.04 MB PDF) [file pone.0003306.s002.pdf]

Figure S3

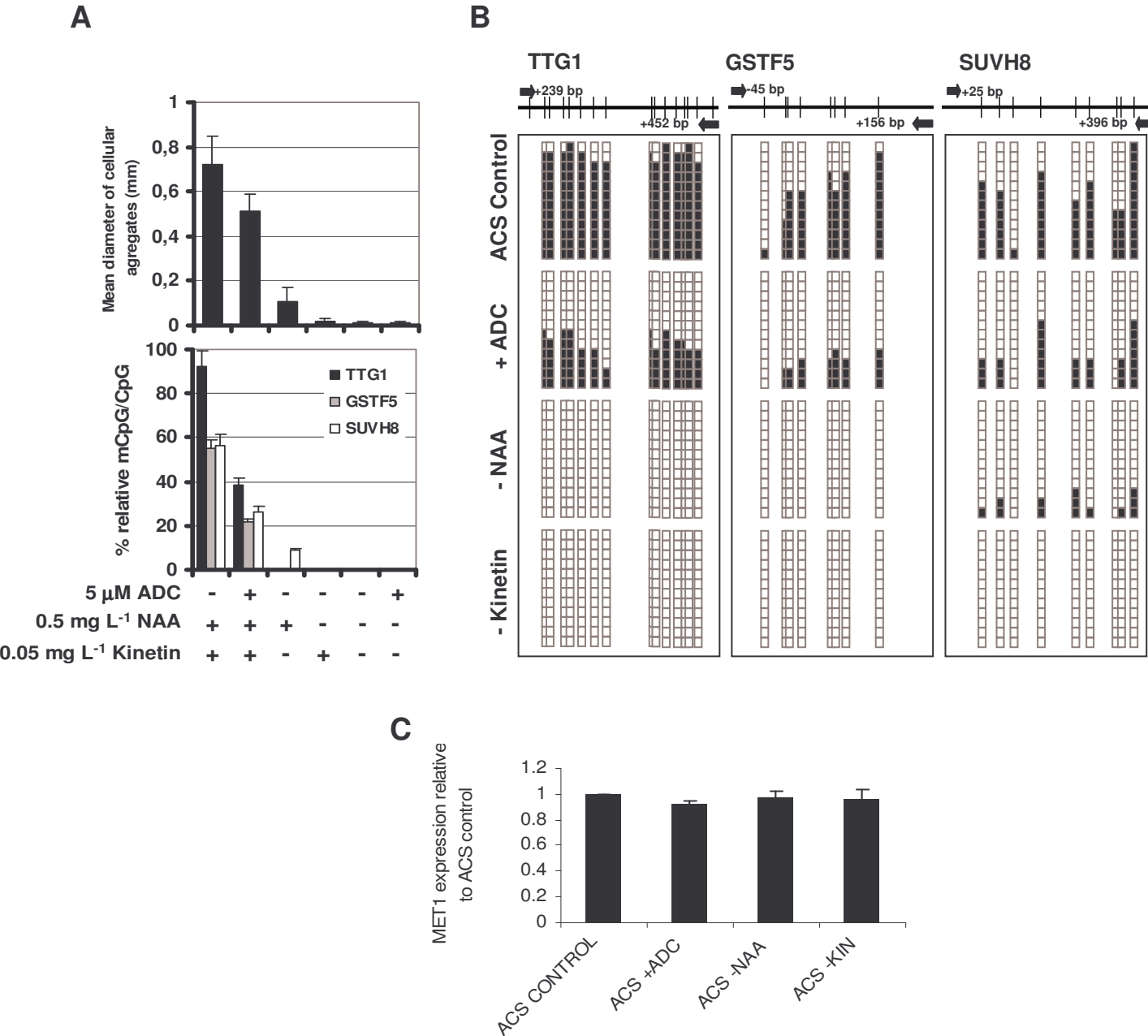

Supplement: Figure S3 — Promoter hypermethylation in cell suspensions is dependent on combined phytohormone action and is associated with histone hypoacetylation and gene repression. Bisulfite genomic sequencing of the TTG1, GSTF5, and SUVH8 promoters in the presence or absence of the demethylating drug ADC, and the phytohormones NAA and kinetin in Arabidopsis cell suspensions. (A) Quantification of growth rates and the relative percentage of methylated CpGs. (B) Schematic representations of the methylation status of each CpG dinucleotide. Black and white dots indicate methylated and unmethylated CpGs, respectively. (C) Analysis of MET1 expression in Arabidopsis cell suspensions (ACS) growing in normal culture medium, in presence or in absence of phytohormones (kinetin or NAA). Transcript levels were analyzed by quantitative RT-PCR and results are expressed as a value relative to the expression in control ACS. (0.04 MB PDF) [file pone.0003306.s003.pdf]

Figure S4

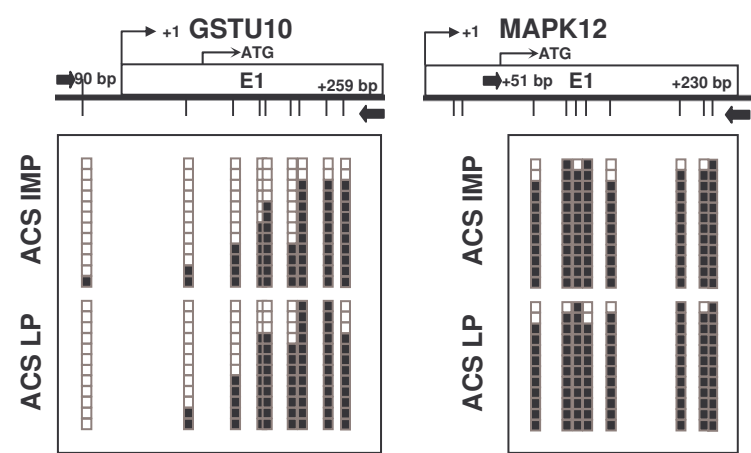

Supplement: Figure S4 — Promoter DNA methylation of MAPK12 and GSTU10 genes was also analyzed after different numbers of passages in Arabidopsis cell suspensions (ACS): IMP, intermediate passages; LP, late passages. Black and white dots indicate methylated and unmethylated CpGs, respectively. (0.02 MB PDF) [file pone.0003306.s004.pdf]

Figure S5

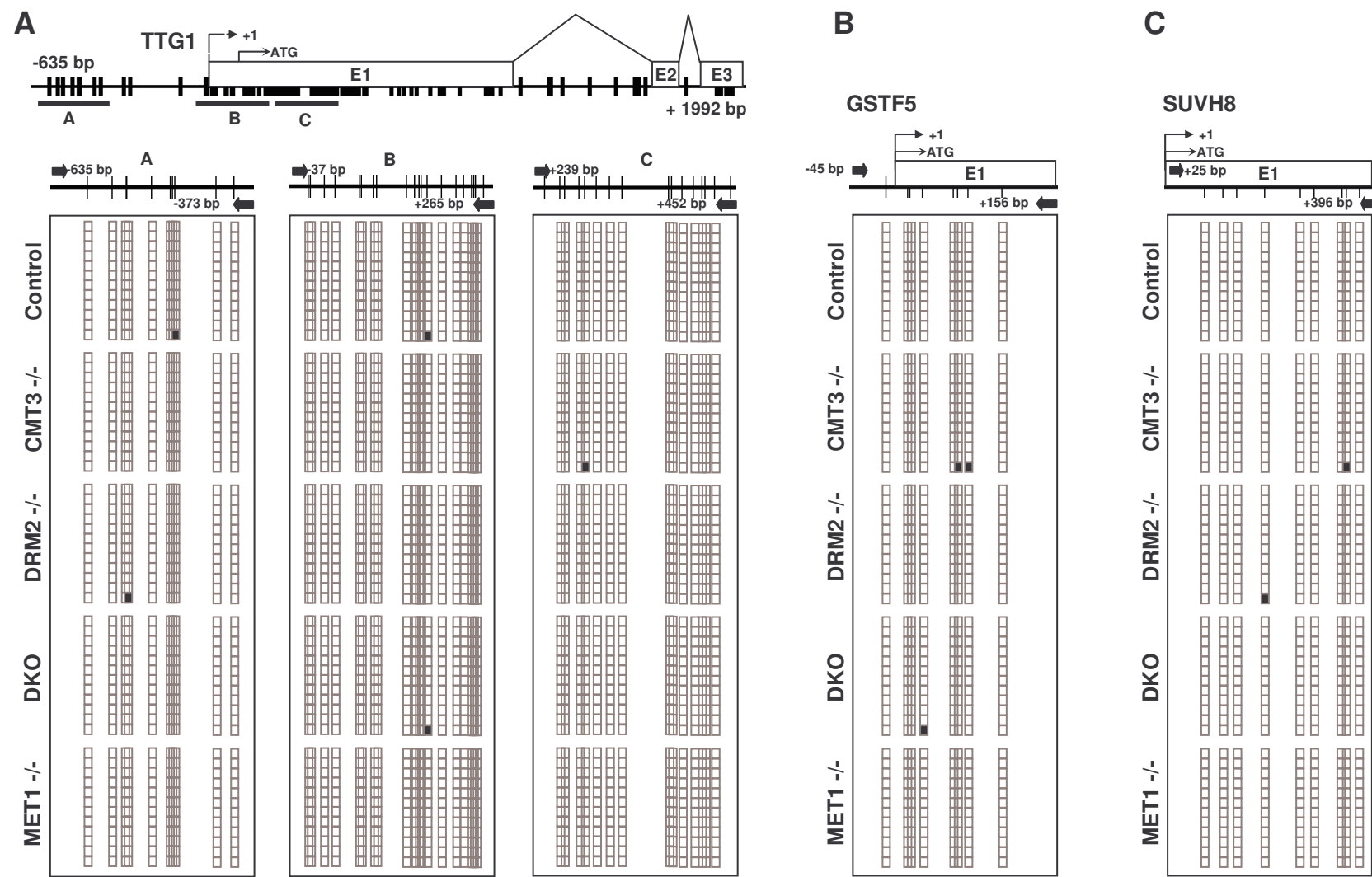

Supplement: Figure S5 — Bisulfite genomic sequencing of twelve individual clones of TTG1 (A), GSTF5 (B), and SUVH8 (C) genes in cell suspensions derived from WT and DNA methyltransferase mutants of Arabidopsis thaliana (L.). Black and white dots indicate methylated and unmethylated CpGs, respectively. (0.03 MB PDF) [file pone.0003306.s005.pdf]

Figure S6

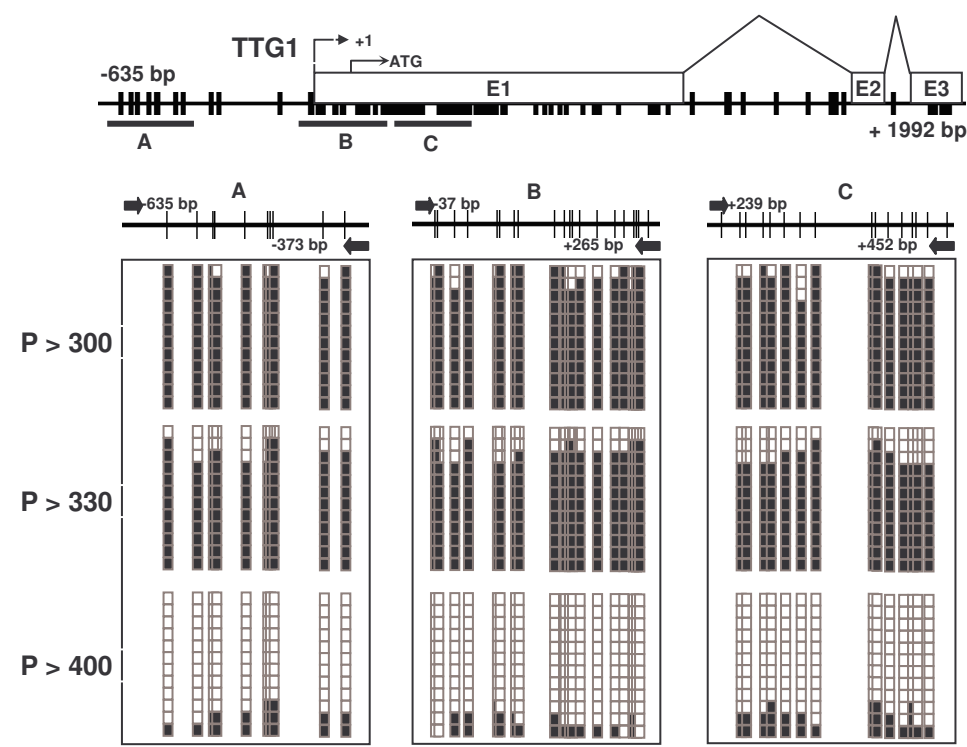

Supplement: Figure S6 — Bisulfite genomic sequencing of twelve clones of the TTG1 (three regions) 5′-regulatory region in Arabidopsis cell suspensions at 300, 330, and 400 passages. Schematic representations of the methylation status of each CpG dinucleotide. Black and white dots indicate methylated and unmethylated CpGs, respectively. (0.03 MB PDF) [file pone.0003306.s006.pdf]

**Figure S7**

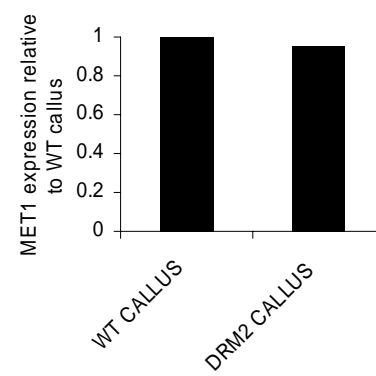

Supplement: Figure S7 — Transcription levels of MET1 in WT callus and drm2 mutant. Transcript levels were analyzed by quantitative RT-PCR and results are expressed as a value relative to the expression in WT callus. (0.01 MB PDF) [file pone.0003306.s007.pdf]
