# Supplementary material for: Promoter DNA Hypermethylation and Gene Repression in Undifferentiated Arabidopsis Cells
Source: PLoS One. 2008 Oct 1;3(10):e3306. doi: 10.1371/journal.pone.0003306 (PMC2556100; doi:10.1371/journal.pone.0003306)
Supplement: Table S1 — Primer sequences and annealing temperatures. (0.02 MB PDF) [file pone.0003306.s008.pdf]

Bisulfite sequencing

Gene	Gene ID	5´primer	3´primer	Tm (ºC)
AGL20	At2g45660	tgaagtttttttgtttttgtttt	aaacaaaaaaaccaaacacatc	58
ANACO41	At2g33480	aaaaaaaaaatttatgaggttttg	cctctrtttttaataaaactcctctt	55
AP2	At2g23340	gtgaatttttaattttttgttttttt	cctaaccaratccttaatctct	55
ARK3	At4g21380	ttattggaaaaggaaagaaagt	aaaccaaatttttatttttcact	56
ATGLR2.5	At5g11210	tgaattggttattttttttaaaat	aaacaatccttatcttaaaacca	56
ATINT4	At4g16480	agggaggtttttttttatttt	cattttattcctttccatttaaa	56
BD	At1g58025	tagtttttggttgttgyaggag	taaattcaaattcactattctcacac	58
BRCT	At4g21070	gagatgagagtttytggtttgg	caaaacaccaatctttacacaaac	59
BT2	At3g48360	ttttttgatgatttatggaag	aaaaaaaaaaaactttaacacatttc	56
BXL1	At5g49360	tagtttggttaaaagtttattggt	attaaaattctcctctttccat	55
BZO2H3	At5g28770	tgttttgataatttgaaattaaaaa	cacaaacaaaattaaatttactctta	58
CACTA(1)	At1g43840	ggagaatatttatgtatggtataggt	aaactatcaaaaaactcatcca	56
CACTA(2)	At2g13160	gtgggaatatttyaggggtttaa	aacttaaccttaccataaaattccc	60
CACTA(3)	At2g12980	gatttttattgtagatytgtttagg	ccaraactaacaaacaactcttc	56
CCD7	At2g44990	aaaaatgttgattaaaatgtttttt	ctaaaaaataaaccrttaaacca	56
CCR4	At3g44260	caaatcataaaccaaagcttcta	aaattaaactcccarataaatcctc	58
CLV1	At1g08590	gaactacttgctatcatacaaattg	taaaaaccaacaaaatctcttattc	57
CYC1	At4g37490	atagttaataaaaagaataagtgggt	ttaatattctcttctctttctctca	55
DC1	At2g44370	tgattttgaattttaaatgagaa	aacaaataatctcttcttcaactta	55
DL1D	At2g44590	tggttttggttaattatattgaa	aaaattatctarttaaaacaaaacct	54
EDA28	At2g34790	tgtttaagggatataytgtttattttt	tcaaactcctctttaaacaaaataa	56
eIF5A	At1g69410	tttgttttttaaattttgttttg	aaatttcaraaaaaaaatattcttc	55
ERF2	At5g47220	gttattttgatttttttttattgtgatat	ctcctcctcctcctaacaaata	58
Fbox	At2g02300	gaatttattagatagatatgagaagt	tcccaacaaccacaaaatt	56
Fimbrin	At2g04750	ttggtgtttttgtttytgattt	ttggtgtttttgtttytgattt	56
GERMIN	At1g18980	tttttttttttgtgatytgtgt	aaaccaatcctctaaaaacaac	56
GSTF5	At1g02940	tttttgtgttatggttttgaatattt	tctttattccatcccattaattaata	54
GSTF7	At1g02930	ataataaagagtatttaagtttggtgg	aaaaactctttcttatattcacca	56
GSTU10	At1g74590	ggaatatttaaagttttgatttttg	ctratttcccatcataaacaaa	56
GTP	At5g54840	tgagtttgtgaatgttaaaaga	aaaaataatcttcccratttaacaa	56
HAP3	At4g14540	gttattaagtttttttttttttttt	ttaattatcttcctcttctctctct	55
HSP81	At5g52640	gatggtaagtgtttttgtaatgatt	tcttcttttaaaataaaaaaaaacca	58
LEA1	At1g32560	agttatagttttggaggattttg	ccaaataaatcacattaattcc	55
Leucine-rich	At1g07390	gatgtgtaagytgtgtggaaa	caaacaaattaataaaccaattc	56
MAPK12	At2g46070	tggagaattaagttytggttt	aatcaaaaaaacttaccaracaa	56
MTase	At4g33110	atttttttttggtatagtaaaatgg	ttatctcttaaatttttttccct	55
MtN21	At4g28040	agggttaattttattttttgaa	tttcttacaaataaatcttaaacca	55
PEX11	At3g47430	aaatttttttttttattttaagtttga	tctcttaaccaaaaacacca	56
PLL4	At2g28890	ataaaagaagttttggatttaaataat	aaaaaaaaaaaaaatttttttttact	55
PsbQ	At3g01440	gattttttttagatyagagagagaga	tatatcttctttactttttccttcac	58
RPT2	At2g30520	agtttattattaaaaagttggtgaaa	aaatttctttaacataaatcccc	56
SEN1	At4g35770	agttatttgttttaatattgggttt	cartaatttccattttcacttc	56
toIB	At4g01870	atatttggttggtgaaaattag	taataccttttaaaaatttccattatc	55
Trehalose	At5g65140	agaaagggattatttatataaagagaa	aaataattaaaaaaaataaaaccaaac	56
TTG1-s1	At5g24520	ggaagtttttygaagattataataa	atcttcrttaatttcccaaaaac	56

TTG1-s2	At5g24520	tgattaattyagagggtttatattta	ttattataatcttcraaaaaacttcc	54
TTG1-s3	At5g24520	tatttatttgtaaggtggagtga	acaaaccaaacttccatacat	56
SUVH8	At2g24740	aatgttatttgatgatggagat	cttcattaacttcataaaatacttctc	56
UDP	At2g43820	gatatgtaaattggttttgttttttt	aaacccccataatcataacc	58

ChIP

Gene	*Gene ID*	*5´primer*	*3´primer*	*Tm (ºC)*
ACTIN	At5g09810	ctcccattcccttctccttc	cgaggacgacccacaatact	63
GSTF5	At1g02940	tcatggttatgctgtccatttc	agcaagtttccggtacatgg	63
SUVH8	At2g24740	ccacgcaacatcagtacctc	gtactaggaccggcatctcg	63
TTG1	At5g24520	tctacgccatggctttctct	gagatctccggaggaaggac	63

RT-PCR

Gene	*Gene ID*	*5´primer*	*3´primer*	*Tm (ºC)*
ACTIN	At5g09810	ctcccattcccttctccttc	cgaggacgacccacaatact	60
GSTF5	At1g02940	tgaccagaagaagccgagtt	cccaggtcagtgttgatgtg	60
SUVH8	At2g24740	tgatgatggagatgctggtc	acccatttttcgaacccttt	60
TTG1	At5g24520	ctcggttctcaacaacagca	cgcacatgacgattatacgg	60

Q-PCR

Gene	*Gene ID*	*5´primer*	*3´primer*	*Tm (ºC)*
ACTIN	At5g09810	tacaacgagcttcgtgttgc	tacatggcagggacattgaa	60
BXL1	At5g49360	ggagcccttgtaaaatgctg	atgaaaacgccattgctctc	60
CCD7	At2g44990	acagccgcagatttgttga	cgaccgtcgactttgtaatg	60
Fimbrin	At2g04750	gccaattcacacaagtggag	tcacagtgacacgaccgaat	60
GSTF5	At1g02940	tgaccagaagaagccgagtt	aagacccccgtctaggaaaa	60
GSTU10	At1g74590	cggaggctacaaagctcatc	gatcttgcaggcgctctatc	60
MAPK12	At2g46070	gcgatacccgaaacaacagt	tcatggtgcggtgataggta	60
SUVH8	At2g24740	gtcgggcttcacagaaacac	actcgtagcagcaggaccat	60
TTG1	At5g24520	ctctacgccatggctttctc	atgtcgatgcggttgttgta	60

Table S1. Primer sequences and annealing temperatures (Tm) for bisulfite sequencing, chromatin immunoprecipitation (ChIP) RT-PCR and Q-PCR reactions.
