# Supplementary material for: Promoter DNA Hypermethylation and Gene Repression in Undifferentiated Arabidopsis Cells
Source: PLoS One. 2008 Oct 1;3(10):e3306. doi: 10.1371/journal.pone.0003306 (PMC2556100; doi:10.1371/journal.pone.0003306)
Supplement: Table S2 — Genetic identification of methylated genes in dedifferentiated Arabidopsis cells. (0.02 MB PDF) [file pone.0003306.s009.pdf]

Probe Set ID	Gene Title	Representative Public ID	Genes selected for mutant:
247228_at	trehalose-6-phosphate phosphatase, putative	At5g65140	CMT3
252411_at	peroxisomal biogenesis factor 11 family protein / PEX11 family protein	At3g47430	DRM2
266600_at	mitogen-activated protein kinase, putative / MAPK, putative (MPK12)	At2g46070	DRM2
245592_at	CCAAT-box binding transcription factor subunit B (NF-YB) (HAP3)	At4g14540	DRM2
267509_at	MADS-box protein (AGL20)	At2g45660	DRM2
245499_at	sugar transporter family protein (ATINT4)	At4g16480	DRM2
248622_at	glycosyl hydrolase family 3 protein(BXL1)	At5g49360	DRM2
253829_at	nodulin MtN21 family protein	At4g28040	DRM2
256464_at	late embryogenesis abundant group 1 domain-containing protein (LEA1)	At1g32560	DRM2
253161_at	senescence-associated protein (SEN1)	At4g35770	DRM2
250415_at	glutamate receptor family protein (GLR2.5)	At5g11210	MET1
260350_at	eukaryotic translation initiation factor 5A, putative / eIF-5A, putative	At1g69410	MET1
255543_at	tolB protein-related	At4g01870	MET1
254416_at	S-locus protein kinase, putative (ARK3)	At4g21380	MET1
252367_at	speckle-type POZ protein-related (BT2)	At3g48360	MET1
253362_s_at	coclaurine N-methyltransferase, putative	At4g33110	MET1
267516_at	signal transducer of phototropic response (RPT2)	At2g30520	MET1
245078_at	AP2 domain-containing transcription factor, putative	At2g23340	MET1
248794_at	ethylene-responsive element-binding factor 2 (ERF2)	At5g47220	MET1
257377_at	protein phosphatase 2C family protein / PP2C family protein (PLL4)	At2g28890	MET1
255794_at	no apical meristem (NAM) family protein (ANACO41)	At2g33480	MET1
267384_at	DC1 domain-containing protein	At2g44370	MET1
267414_at	FAD-binding domain-containing protein (EDA28)	At2g34790	MET1
248132_at	GTP-binding family protein	At5g54840	MET1
248332_at	heat shock protein 81-1 (HSP81-1) / heat shock protein 83 (HSP83)	At5g52640	MET1
260567_at	UDP-glucoronosyl/UDP-glucosyl transferase family protein	At2g43820	MET1
247913_at	hypothetical protein	At5g57510	MET1
259478_at	germin-like protein, putative	At1g18980	MET1
260225_at	glutathione S-transferase, putative (GSTU10)	At1g74590	MET1
261070_at	leucine-rich repeat family protein	At1g07300	MET1
262119_s_at	glutathione S-transferase, putative (GSTF7)	At1g02930	MET1

Table S2. Genetic identification of methylated genes in *Arabidopsis* callus. Candidate genes obtained by Affymetrix GeneChip technology with *met1, drm2* and *cmt3* mutants. Genes were selected using the criteria described in the results section.
