# Supplementary material for: Promoter DNA Hypermethylation and Gene Repression in Undifferentiated Arabidopsis Cells
Source: PLoS One. 2008 Oct 1;3(10):e3306. doi: 10.1371/journal.pone.0003306 (PMC2556100; doi:10.1371/journal.pone.0003306)
Supplement: Table S3 — List of candidate genes selected for validation with bisulfite sequencing analysis. (0.03 MB PDF) [file pone.0003306.s010.pdf]

Gene ID	Gene	Process	References
At1g02930	*GSTF7 (ATGSTF6)(**)*	Gluatione S-Transferase	Moeder et al. 2007. Plant Mol Biol 63(2):273-287
At1g02940	*GSTF5 (ATGSTF5) (**)*	Gluatione S-Transferase	Wagner et al., 2002. Plant Mol Biol. 49(5):515-532.
At1g07390	*ATRLP1*	Signal transduction	Kobe B, Kajava AV.2001. Curr Opin Struct Biol 11(6):725-732.
At1g08590	*CLV1*	Protein amino acid phosphorylation, signaling pathway	Duarte et al., 2006. Mol. Biol. Evol. 23(2):469-478.
At1g18980	*GERMIN*	Manganese ion binding	Christensen et al. 2004. Mol Plant Microbe Interact 17(1): 109-117*
At1g32560	*LEA1*	Embryonic development ending in seed dormancy	Hundertmark and Hincha. 2008. BMC Genomics 9:118.
At1g58025	*BD*	DNA binding	Chua et al. 2005. Genes Dev19(18): 2245-2254*
At1g69410	*EIF5A*	Translational initiation	Balabanov et al. 2007. Blood 109(4):1701-1711*.
At1g74590	*GSTU10 (ATGSTU10) (**)*	Gluatione S-Transferase	Wagner et al., 2002. Plant Mol Biol. 49(5):515-532.
At2g02300	*Fbox (ATPP2-B5) (**)*	Carbohydrate binding	Dinant et al. 2003. Plant Physiol. 131(1):114-128.
At2g04750	*Fimbrin*	Actin binding	Reichl et al. 2008. Curr Biol 18(7): 471- 480.
At2g23340	*AP2*	Regulation of transcription	Pre et al. 2008. Plant Physiol, in press.
At2g24740	*SUVH8 (SDG21) (**)*	Regulation of gene expression, epigenetics	Baumbusch et al., 2001. Nucleic Acids Research 29(21):4319-33
At2g28890	*PLL4*	Leaf development	Song et al. 2005. Dev Biol. 285(1):272-284.
At2g30520	*RPT2*	Protein binding, phototropism	Inada et al. 2004. Plant Cell 16(4):887- 896.
At2g33480	*ANACO41*	Multicellular organismal development	Yoo et al. 2007. PLoS ONE 2(7):e642.
At2g34790	*EDA28*	Embryonic development ending in seed dormancy, polar nucleus fusion	Pagnussat et al. 2005. Development 132(3):603-614.
At2g43820	*UDP*	UDP-glucosyltransferase	Sepúlveda-Jiménez et al. 2005. J Exp Bot 56(412):605-611*.
At2g44370	*DC1*	Protein binding	Shinya et al. 2007. Plant Cell Physiol. 48(10):1404-1413*.
At2g44590	*DL1D (ADL1D) (**)*	Growth and development	Kang et al. 2003. Plant Cell. 15(4):899-913.
At2g44990	*CCD7*	Secondary shoot formation	Stirnberg et al. 2007. Plant J 50(1):80-94.
At2g45660	*AGL20*	Positive regulation of flowering	Liu et al. 2007. Development 134(10):1901-1910.
At2g46070	*MAPK12 (ATMPK12) (**)*	Signal transduction	Hamel et al., 2006. Trends Plant Sci.11(4):192-198.
At3g01440	*PsbQ*	Light reaction	Zybailov et al. 2008. PLoS ONE 3(4):e1994
At3g44260	*CCR4*	RNA modification	Prévôt et al. 2001. J Biol Chem 276(13): 9640-9648*
At3g47430	*PEX11*	Peroxisome organization and biogenesis	Orth et al. 2007. Plant Cell 19(1):333-350.
At3g48360	*BT2*	Positive regulation of telomerase activity	Ren et al. 2007. Plant Cell 19(1): 23-31.
At4g01870	*tolB*	Translocation domain	Mueller et al. 2008. Plant Cell 20: 768-785*

At4g14540	*HAP3*	Regulation of transcription	Lee et al. 2003. Proc Natl Acad Sci U S A. 100(4):2152-2156.
At4g16480	*ATINT4*	Carbohydrate transport	Schneider et al. Plant Physiol 145(4):1395-1407.
At4g21070	*BRCT (ATBRCA1) (**)*	DNA repair	Reidt et al., 2006.EMBO J. 25(18):4326-4337.
At4g21380	*ARK3*	Protein amino acid phosphorylation	Pastuglia et al. 2002. Gene 282(1-2): 215-225.
At4g28040	*MtN21*	Amino acid transport	Busov et al. 2004. Planta 218(6):916-927*.
At4g33110	*Mtase*	N-methyltransferase	Liscombe and Facchini. 2007. J Biol Chem 282(20):14741-14751*.
At4g35770	*SEN1*	Aging	Schenk et al. 2005. Plant Physiol Biochem 43(10-11): 997-1005.
At4g37490	*CYC1*	Regulation of cell growth and cell cycle	Kirik et al., 2007. Plant Cell. 19(10):3100-3110.
At5g11210	*AGLR2.5*	Response to light stimulus	Shin et al. 2008. Neuropharmacology, in press.
At5g24520	*TTG1*	Cell fate and differentiation	Bouyer et al., 2008. PLoS Biol. 6: e141.
At5g28770	*BZO2H3*	Regulation of transcription	Jakoby et al. 2002. Trends Plant Sci 7(3):106-111.
At5g47220	*ERF2*	Positive regulation of transcription	Nakano et al. 2006. Plant Physiol. 140(2):411-432.
At5g49360	*BXL1*	Secondary wall thickening	Zybailov et al. 2008. PLoS ONE 3(4):e1994.
At5g52640	*HSP81*	Heat shock	Takahashi et al. 2003. Proc Natl Acad Sci U S A.100(20):11777-11782.
At5g54840	*GTP*	Small GTPase mediated signal transduction	Weirich et al. 2008. Nat Rev Mol Cell Biol 9(6):478-489*.
At5g65140	*trehalose*	trehalose biosynthetic process	Chary et al. 2008. Plant Physiol 146(1): 97-107*

Table S3. List of candidate genes selected for the validation with the bisulfite sequencing analysis. Genes were obtained from both the pharmacological and genetic approaches. Then, we selected candidates attending to their functional role as described in the literature.

** member of the same family.*
***nomenclature from genome annotation: Build 7.0.*

References Table S3

1. Balabanov S, Gontarewicz A, Ziegler P, Hartmann U, Kammer W, et al. (2007) Hypusination of eukaryotic initiation factor 5A (eIF5A): a novel therapeutic target in BCR-ABL-positive leukemias identified by a proteomics approach. Blood 109: 1701-1711.
2. Baumbusch LO, Thorstensen T, Krauss V, Fischer A, Naumann K, et al. (2001) The Arabidopsis thaliana genome contains at least 29 active genes encoding SET domain proteins that can be assigned to four evolutionarily conserved classes. Nucleic Acids Res 29: 4319-4333.
3. Bouyer D, Geier F, Kragler F, Schnittger A, Pesch M, et al. (2008) Two-dimensional patterning by a trapping/depletion mechanism: the role of TTG1 and GL3 in Arabidopsis trichome formation. PLoS Biol 6: e141.
4. Busov VB, Johannes E, Whetten RW, Sederoff RR, Spiker SL, et al. (2004) An auxin-inducible gene from loblolly pine (Pinus taeda L.) is differentially expressed in mature and juvenile-phase shoots and encodes a putative transmembrane protein. Planta 218: 916-927.

5. Chary SN, Hicks GR, Choi YG, Carter D, Raikhel NV (2008) Trehalose-6-phosphate synthase/phosphatase regulates cell shape and plant architecture in Arabidopsis. Plant Physiol 146: 97-107.
6. Christensen AB, Thordal-Christensen H, Zimmermann G, Gjetting T, Lyngkjaer MF, et al. (2004) The germinlike protein GLP4 exhibits superoxide dismutase activity and is an important component of quantitative resistance in wheat and barley. Mol Plant Microbe Interact 17: 109-117.
7. Chua YL, Channeliere S, Mott E, Gray JC (2005) The bromodomain protein GTE6 controls leaf development in Arabidopsis by histone acetylation at ASYMMETRIC LEAVES1. Genes Dev 19: 2245-2254.
8. Dinant S, Clark AM, Zhu Y, Vilaine F, Palauqui JC, et al. (2003) Diversity of the superfamily of phloem lectins (phloem protein 2) in angiosperms. Plant Physiol 131: 114-128.
9. Duarte JM, Cui L, Wall PK, Zhang Q, Zhang X, et al. (2006) Expression pattern shifts following duplication indicative of subfunctionalization and neofunctionalization in regulatory genes of Arabidopsis. Mol Biol Evol 23: 469-478.
10. Hamel LP, Nicole MC, Sritubtim S, Morency MJ, Ellis M, et al. (2006) Ancient signals: comparative genomics of plant MAPK and MAPKK gene families. Trends Plant Sci 11: 192-198.
11. Hundertmark M, Hincha DK (2008) LEA (late embryogenesis abundant) proteins and their encoding genes in Arabidopsis thaliana. BMC Genomics 9: 118.
12. Inada S, Ohgishi M, Mayama T, Okada K, Sakai T (2004) RPT2 is a signal transducer involved in phototropic response and stomatal opening by association with phototropin 1 in Arabidopsis thaliana. Plant Cell 16: 887-896.
13. Jakoby M, Weisshaar B, Droge-Laser W, Vicente-Carbajosa J, Tiedemann J, et al. (2002) bZIP transcription factors in Arabidopsis. Trends Plant Sci 7: 106-111.
14. Kang BH, Busse JS, Bednarek SY (2003) Members of the Arabidopsis dynamin-like gene family, ADL1, are essential for plant cytokinesis and polarized cell growth. Plant Cell 15: 899-913.
15. Kirik V, Schrader A, Uhrig JF, Hulskamp M (2007) MIDGET unravels functions of the Arabidopsis topoisomerase VI complex in DNA endoreduplication, chromatin condensation, and transcriptional silencing. Plant Cell 19: 3100-3110.
16. Kobe B, Kajava AV (2001) The leucine-rich repeat as a protein recognition motif. Curr Opin Struct Biol 11: 725-732.
17. Lee H, Fischer RL, Goldberg RB, Harada JJ (2003) Arabidopsis LEAFY COTYLEDON1 represents a functionally specialized subunit of the CCAAT binding transcription factor. Proc Natl Acad Sci U S A 100: 2152-2156.
18. Liscombe DK, Facchini PJ (2007) Molecular cloning and characterization of tetrahydroprotoberberine cis-N-methyltransferase, an enzyme involved in alkaloid biosynthesis in opium poppy. J Biol Chem 282: 14741-14751.
19. Liu C, Zhou J, Bracha-Drori K, Yalovsky S, Ito T, et al. (2007) Specification of Arabidopsis floral meristem identity by repression of flowering time genes. Development 134: 1901-1910.
20. Moeder W, Del Pozo O, Navarre DA, Martin GB, Klessig DF (2007) Aconitase plays a role in regulating resistance to oxidative stress and cell death in Arabidopsis and Nicotiana benthamiana. Plant Mol Biol 63: 273-287.
21. Mueller S, Hilbert B, Dueckershoff K, Roitsch T, Krischke M, et al. (2008) General detoxification and stress responses are mediated by oxidized lipids through TGA transcription factors in Arabidopsis. Plant Cell 20: 768-785.
22. Nakano T, Suzuki K, Fujimura T, Shinshi H (2006) Genome-wide analysis of the ERF gene family in Arabidopsis and rice. Plant Physiol 140: 411-432.
23. Orth T, Reumann S, Zhang X, Fan J, Wenzel D, et al. (2007) The PEROXIN11 protein family controls peroxisome proliferation in Arabidopsis. Plant Cell 19: 333-350.
24. Pastuglia M, Swarup R, Rocher A, Saindrenan P, Roby D, et al. (2002) Comparison of the expression patterns of two small gene families of S gene family receptor kinase genes during the defence response in Brassica oleracea and Arabidopsis thaliana. Gene 282: 215-225.
25. Pre M, Atallah M, Champion A, De Vos M, Pieterse CM, et al. (2008) The AP2/ERF-domain transcription factor ORA59 integrates jasmonic acid and ethylene signals in plant defense. Plant Physiol.

26. Prevot D, Morel AP, Voeltzel T, Rostan MC, Rimokh R, et al. (2001) Relationships of the antiproliferative proteins BTG1 and BTG2 with CAF1, the human homolog of a component of the yeast CCR4 transcriptional complex: involvement in estrogen receptor alpha signaling pathway. J Biol Chem 276: 9640-9648.
27. Reichl EM, Ren Y, Morphew MK, Delannoy M, Effler JC, et al. (2008) Interactions between myosin and actin crosslinkers control cytokinesis contractility dynamics and mechanics. Curr Biol 18: 471-480.
28. Reidt W, Wurz R, Wanieck K, Chu HH, Puchta H (2006) A homologue of the breast cancer-associated gene BARD1 is involved in DNA repair in plants. Embo J 25: 4326-4337.
29. Ren S, Mandadi KK, Boedeker AL, Rathore KS, McKnight TD (2007) Regulation of telomerase in Arabidopsis by BT2, an apparent target of TELOMERASE ACTIVATOR1. Plant Cell 19: 23-31.
30. Schenk PM, Kazan K, Rusu AG, Manners JM, Maclean DJ (2005) The SEN1 gene of Arabidopsis is regulated by signals that link plant defence responses and senescence. Plant Physiol Biochem 43: 997-1005.
31. Schneider S, Schneidereit A, Udvardi P, Hammes U, Gramann M, et al. (2007) Arabidopsis INOSITOL TRANSPORTER2 mediates H+ symport of different inositol epimers and derivatives across the plasma membrane. Plant Physiol 145: 1395-1407.
32. Shin SS, Martino JJ, Chen S (2008) Metabotropic glutamate receptors (mGlus) and cellular transformation. Neuropharmacology.
33. Shinya T, Galis I, Narisawa T, Sasaki M, Fukuda H, et al. (2007) Comprehensive analysis of glucan elicitor-regulated gene expression in tobacco BY-2 cells reveals a novel MYB transcription factor involved in the regulation of phenylpropanoid metabolism. Plant Cell Physiol 48: 1404-1413.
34. Song SK, Clark SE (2005) POL and related phosphatases are dosage-sensitive regulators of meristem and organ development in Arabidopsis. Dev Biol 285: 272-284.
35. Stirnberg P, van De Sande K, Leyser HM (2002) MAX1 and MAX2 control shoot lateral branching in Arabidopsis. Development 129: 1131-1141.
36. Takahashi A, Casais C, Ichimura K, Shirasu K (2003) HSP90 interacts with RAR1 and SGT1 and is essential for RPS2-mediated disease resistance in Arabidopsis. Proc Natl Acad Sci U S A 100: 11777-11782.
37. Wagner U, Edwards R, Dixon DP, Mauch F (2002) Probing the diversity of the Arabidopsis glutathione S-transferase gene family. Plant Mol Biol 49: 515-532.
38. Weirich CS, Erzberger JP, Barral Y (2008) The septin family of GTPases: architecture and dynamics. Nat Rev Mol Cell Biol 9: 478-489.
39. Yoo SY, Kim Y, Kim SY, Lee JS, Ahn JH (2007) Control of flowering time and cold response by a NAC-domain protein in Arabidopsis. PLoS ONE 2: e642.
40. Zybailov B, Rutschow H, Friso G, Rudella A, Emanuelsson O, et al. (2008) Sorting signals, N-terminal modifications and abundance of the chloroplast proteome. PLoS ONE 3: e1994.
