# Supplementary material for: Promoter DNA Hypermethylation and Gene Repression in Undifferentiated Arabidopsis Cells
Source: PLoS One. 2008 Oct 1;3(10):e3306. doi: 10.1371/journal.pone.0003306 (PMC2556100; doi:10.1371/journal.pone.0003306)
Supplement: Table S4 — List of Arabidopsis thaliana genes upregulated after treatment with ADC. (0.02 MB PDF) [file pone.0003306.s011.pdf]

Probe Set ID	Representative Public ID	Gene Title	MEAN WT	MEAN ADC	MEAN FC
258956_at	At3g01440	oxygen evolving enhancer 3 (PsbQ) family protein	1	24.2	24.2
245865_at	At1g58025	DNA-binding bromodomain-containing protein	6.2	67.55	10.89516129
249739_at	At5g24520	transparent testa glabra 1 protein (TTG1)	7.15	56.95	7.965034965
266179_at	At2g02300	F-box family protein / SKP1 interacting partner 3-related	4.45	26.1	5.865168539
264804_at	At1g08590	CLAVATA1 receptor kinase (CLV1)	10	28.4	2.84
266879_at	At2g44590	dynamin-like protein D (DL1D)	12.1	31.6	2.611570248
252679_at	At3g44260	CCR4-NOT transcription complex protein, putative	185.9	484.5	2.606239914
254443_at	At4g21070	BRCT domain-containing protein / zinc finger (C3HC4-type RING finger) family protein (BRCA1)	67.05	167.45	2.497390007
262103_at	At1g02940	glutathione S-transferase, putative	8.5	18.45	2.170588235
263317_s_at	At2g24740	SET domain-containing protein (SUVH7/SUVH8)	146.15	301	2.059527882
253051_at	At4g37490	G2/mitotic-specific cyclin (CYC1) / B-like cyclin (CYC1)	263.75	531.2	2.014028436
245925_at	At5g28770	bZIP transcription factor family protein	23.1	61.6	2.666666667
263642_at	At2g04750	fimbrin	8.65	23.1	2.670520231
266129_at	At2g44990	more axillary growth 3 (CCD7)	13.05	28.55	2.187739464

Table S4. List of *Arabidopsis thaliana* candidate genes obtained with the pharmacologic approach (treatment of *Arabidopsis* cell suspensions with the demethylating drug ADC). The genes were selected according to the two-step criteria described in the results section.
