# Supplementary material for: Promoter DNA Hypermethylation and Gene Repression in Undifferentiated Arabidopsis Cells
Source: PLoS One. 2008 Oct 1;3(10):e3306. doi: 10.1371/journal.pone.0003306 (PMC2556100; doi:10.1371/journal.pone.0003306)
Supplement: Table S5 — Sequence context of some of the DNA methylation found in Arabidopsis cell suspensions and leaves. (0.01 MB PDF) [file pone.0003306.s012.pdf]

Sequence type	Locus	Fragment length (bp)	^{m}CG			^{m}CCG			^{m}CAG			^{m}CTG		
			Nº targets	% methylated*		Nº targets	% methylated		Nº targets	% methylated		Nº targets	% methylated	
				Leaf	ACS		Leaf	ACS		Leaf	ACS		Leaf	ACS
Promoters	*TTG1*	213	16	2.1	92.2	8	0	0	1	0	0	0	0	0
	GSTF5	304	8	1.04	55.2	1	0	0	1	0	0	1	0	0
	SUVH8	371	9	3.70	56.5	7	0	0	4	0	0	7	0	0
Repeats	*CACTA1*	159	5	73.1	68.3	1	66.6	0	3	66.6	66.6	6	65.2	62.5
	CACTA2	170	8	90.0	83.3	2	33.3	18.8	3	38.8	33.3	7	64.3	37.1
	CACTA3	263	11	98.5	63.6	2	75.0	0	6	38.4	8.3	4	77.8	16.6

Table S5. Sequence context of the DNA methylation found in *Arabidopsis* cell suspensions and leaves. Data obtained from bisulfite sequencing of 12 individual clones per sequence and sample.
